# Calibrating Fluorescence Microscopy With 3D-Speckler (3D Fluorescence Speckle Analyzer)

**DOI:** 10.21769/BioProtoc.5051

**Published:** 2024-08-20

**Authors:** Chieh-Chang Lin, Aussie Suzuki

**Affiliations:** 1 McArdle Laboratory for Cancer Research, Department of Oncology, University of Wisconsin-Madison, Madison, WI, USA; 2 Carbone Comprehensive Cancer Center, University of Wisconsin-Madison, Madison, WI, USA

**Keywords:** Fluorescence microscopy, PSF, Resolution, Calibration, Confocal microscopy, Super-resolution microscopy

## Abstract

Fluorescence microscopy has been widely accessible and indispensable in cell biology research. This technique enables researchers to label targets, ranging from individual entities to multiple groups, with fluorescent markers. It offers precise determinations of localization, size, and shape, along with accurate quantifications of fluorescence signal intensities. Furthermore, an ideal fluorescence microscope can achieve approximately 250 nm in lateral and 600 nm in axial resolution. Despite its integral role in these measurements, the calibration of fluorescence microscopes is often overlooked. This protocol introduces the use of 3D-Speckler (3D fluorescence speckle analyzer), a semi-automated software tool we have recently developed, for calibrating fluorescence microscopy. Calibration of fluorescence microscopy includes determining resolution limits, validating accuracy in size measurements, evaluating illumination flatness, and determining chromatic aberrations. 3D-Speckler is user-friendly and enables precise quantification of fluorescence puncta, including nanoscale 2D/3D particle size, precise locations, and intensity information. By utilizing multispectral fluorescence beads of known sizes alongside 3D-Speckler, the software can effectively calibrate imaging systems. We emphasize the importance of routine calibration for imaging systems to maintain their integrity and reproducibility, ensuring accurate quantification. This protocol provides a detailed step-by-step guide on using 3D-Speckler to calibrate imaging systems.

Key features

• Semi-automated particle detection.

• Accurate three-dimensional measurement of fluorescent particle sizes.

• High-precision three-dimensional localization of fluorescent particles.

• Precision analysis of point spread function and chromatic aberration in fluorescence microscopy.

## Background

Fluorescence microscopy serves as an essential tool in a variety of research fields, offering the flexibility to analyze specimens ranging from in vitro to in vivo, with resolutions spanning from micrometers to nanometers [1,2]. An advantage of this technique is the ability to detect individual fluorescent targets and either simultaneously or sequentially visualize multiple fluorescent markers. Applications include the characterization of fluorescently labeled purified proteins, as well as the examination of proteins and genes within 2D/3D cell culture systems, tissues, and whole organisms. Fluorescence microscopy is utilized in various in vitro studies, such as investigating the liquid–liquid phase separation of proteins using fluorescently labeled purified proteins [3,4], examining spindle formation through droplet assays [5], and employing fluorescence resonance energy transfer (FRET) to analyze protein–protein interactions [6] and measure mechanical tension [7,8]. Its utility in developmental biology extends to research across a variety of models, including plants, *Drosophila, Xenopus, C. elegans*, zebrafish, and mice [9]. Moreover, fluorescence microscopy is a cornerstone in cell biology research, enabling the investigation of cellular processes in organisms from yeast to humans, across both 2D and 3D culture systems, as well as in living and fixed tissue samples [10,11]. This technique allows researchers to accurately determine spatial locations, relative localizations, dimensions, and shapes at the nanoscale. For instance, it has been demonstrated that standard confocal microscopy can achieve <5 nm precision in determining 2D/3D protein architecture within cells using a dual-color fluorescence approach [12,13]. Furthermore, fluorescence microscopy excels in quantifying protein copy numbers and assessing relative protein concentrations with high accuracy [14–16]. The resolution of fluorescence microscopy is constrained by the point spread function (PSF), which represents the 3D diffraction pattern of light emitted from an infinitely small point source [17]. An ideal fluorescence microscope, equipped with a high numerical aperture (NA) objective lens (NA > 1.4) and a high-resolution camera, can achieve resolutions of approximately 250 nm laterally and 600 nm axially [18,19]. However, the maximum resolution is also contingent on the signal-to-noise ratio (SNR) of the specimens. These resolution limits can be enhanced without altering the optical setup through expansion microscopy (ExM), which physically expands specimens without changing the optics setup, or through post-imaging processing techniques, such as deconvolution [18,20,21]. Additionally, ongoing developments in super-resolution microscopy significantly improve resolutions compared to conventional fluorescence microscopes, enabling the further detailed study of nano-scale cellular structures [1,22–24].

All fluorescence microscopes have the potential to achieve their theoretical resolution maximum and quantitative accuracy. However, this potential heavily relies on the components and the conditions under which microscopy is conducted. To understand the current conditions of the imaging system, it is critical to routinely perform calibrations. We have recently developed the 3D-Speckler software, which enables precise quantification of fluorescence puncta in both biological and non-biological samples [18]. Utilizing fluorescence beads of known sizes in conjunction with 3D-Speckler allows for the comprehensive evaluation of resolution limits, size quantification accuracy, chromatic aberration determination, and the flatness of illumination produced by imaging systems. The aim of this article is to present a comprehensive guide on utilizing the 3D-Speckler software for calibrating imaging systems. This guide is intended for a broad audience interested in fluorescence microscopy, rather than experts in optics and mathematics. Consequently, this protocol minimizes the explanation of the coding and mathematical principles behind 3D-Speckler. For detailed information, please refer to our original manuscript [18]. Briefly, 3D-Speckler identifies fluorescence particles primarily based on their relative signal intensities against the background and determines particle sizes through a full-width-at-half-maximum (FWHM) calculation based on a 2D or 3D Gaussian profile fitted to the intensity profile. The intensity measurements for fluorescence beads can be performed either without background correction or with a local background correction for each bead [14]. 3D-Speckler can determine the center of fluorophores with ~2 nm accuracy, which allows it to accurately identify chromatic aberrations, an unavoidable image distortion that occurs between different wavelengths, by measuring the distance between the centers of fluorophores with different wavelengths within a single bead. This process is designed to evaluate their current performance and ensure their integrity, accuracy, and reproducibility in quantification tasks.

## Materials and reagents

1. TetraSpeck Fluorescent Microspheres Size kit (Thermo Fisher Scientific, catalog number: T14792) (see **General notes 1–2**)
2. Appropriate immersion medium [oil (e.g., Nikon, Type F), water (Mili-Q water), or silicone (e.g., Nikon, silicone immersion oil)] for the objectives you wish to calibrate

## Equipment

1. Any type of fluorescence microscope (e.g., Nikon, model: Nikon-Ti2)
  Example microscope setting: a Nikon Ti-2 inverted microscope equipped with a Yokogawa SoRa-W1 super-resolution spinning-disc confocal installed uniformizer, a high-resolution Hamamatsu Flash V3 CMOS camera, 4-line lasers (405, 488, 561, 640, 100 mW power), and a 60× or a 100× NA 1.4 oil objective
2. Image analysis PC [e.g., Dell, model: Precision 5820 Tower with Intel Core i7-9800X (3.8 GHz), Windows 10 Pro 64, 64 GB 2666 MHz DDR4, and Radeon Pro WX5100 8 GB]

## Software and datasets

1. MATLAB (MathWorks, R2019-b and above, 10/1/2018, license required) (see **General note 3**)
2. 3D-Speckler (https://github.com/suzukilabmcardle/3D-Speckler) (see **General note 4**) (07/20/2024, publicly available)
3. The source code, bfmatlab, user manual, and example test images are available at the following link: https://drive.google.com/drive/u/3/folders/1jKqiYFm31cJ0VVhGhLFhRuLI_ZiqmRjF (07/20/2024, publicly available)
4. Microscope control software (example: Nikon NIS Element, 04/05/2018, license required for NIS Element)

## Procedure

This protocol mainly focuses on describing in detail the 3D-Speckler interface features for microscope calibration and thus offering a comprehensive, step-by-step workflow to operate it.

- **Obtaining images of fluorescent beads**
  For calibration with 3D-Speckler, we recommend utilizing fluorescent beads of at least two different sizes: one smaller than the point spread function (PSF) of your imaging system (less than 200 nm) and one larger (for example, 250 nm, 500 nm, or 1 μm). Smaller beads will be used to determine the lateral resolution limit of your system, which is equivalent to its PSF. Larger beads will be used to evaluate whether your system can accurately measure the size of beads larger than the PSF. In this protocol, we used TetraSpeck beads of 100 and 500 nm. 3D-Speckler can be used for both 2D and 3D images of fluorescence beads obtained from any fluorescence microscope. Additionally, 3D images can be utilized for 2D calibration (see details in our original manuscript [18]). Axial size measurement and 3D chromatic aberration correction require 3D stack images.
  1. Power on the microscope system and select the objectives you plan to use for calibration.
  2. Place a slide with TetraSpeck beads (100 nm) on the microscope stage.
  3. Locate a field of view (FOV) that contains TetraSpeck beads. We recommend finding a FOV containing fluorescence beads that are evenly distributed and not aggregated or too crowded. This helps minimize the difficulty and errors in identifying individual beads and taking measurements. Then, finely adjust the focus, the camera's exposure time, and the light source's power for optimal imaging (see **General note 5**).
  4. (Optional) Set the z-range to encompass the entire depth of the beads, ensuring that the interval between each step is less than 200 nm (see **General note 6**).
  5. Acquire images at the wavelengths you wish to calibrate. It is recommended to capture images at different wavelengths at the same z-plane for chromatic aberration measurements.
  6. Repeat steps A2–A5 at several different locations on the slide to ensure thorough calibration (see **General note 7**).
  7. Using a slide with larger TetraSpeck beads (for example, 500 nm), repeat steps A2–A6 (Figure 1).
    **Figure 1.**
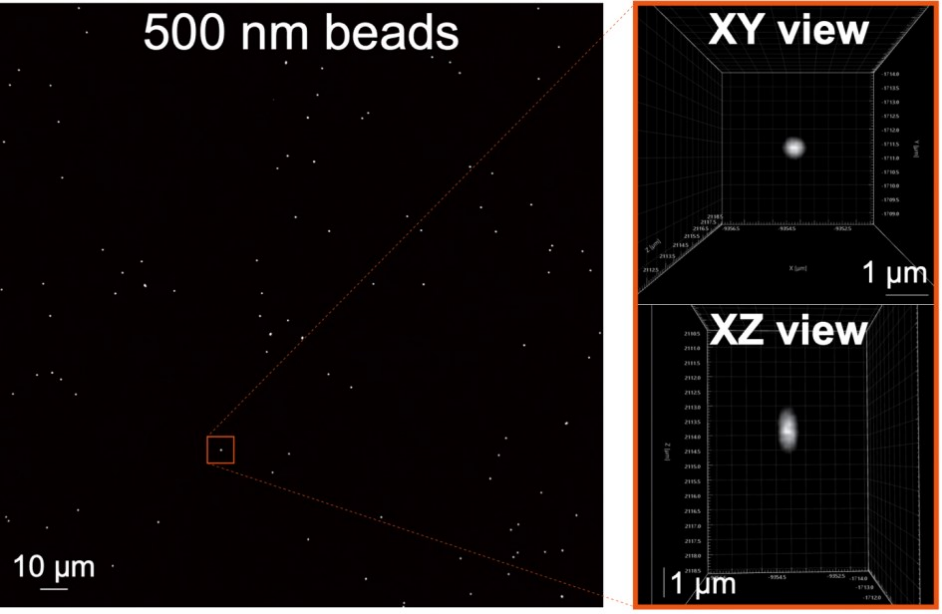
Example of a TetraSpeck bead image. The 500 nm TetraSpeck beads were imaged using a 60× objective lens. The full field of view (FOV) image is displayed on the left. On the right, the XY and XZ views of a single 500 nm bead from the left image are presented. Visualization of XY and XZ views using Imaris software (Andor).
- **Workflow of 3D-Speckler**
  1. 3D-Speckler interface features
Figure 2 displays the primary user interface of 3D-Speckler. A detailed description of each module is listed below.
    - *Open File*: Enables the import of new image files (e.g., *.stk, *.nds, *.tif) for analysis (see **General note 8**).
    - *Import Data*: Users can import data previously analyzed for further visualization (*.xlsx, *.xls).
    - *Import CA (chromatic aberration) Calibration*: Facilitates the import of polynomial calibration surfaces or affine transformation calibrations to correct chromatic aberrations.
    - *Analysis Options*: Offers several options for local background correction, exclusion of overlapping particles, and by-size particle filtering.
    - *Bounding Box Buffer*: Users can adjust the bounding box padding for 2D/3D analysis, but the default value (0.3) should suffice for most fluorescence bead analyses.
    - *Particle Size Filter*: Allows users to filter particles for analysis by setting a range of lateral pixel sizes.
    - *Commit Current ROI (Region of Interest):* Enables the analysis of selected areas within a larger image, with the option to reset the ROI to the original image dimensions.
    - *Channel Choice*: Provides options for users to select channels to analyze or visualize.
    - *Image Viewer*: Visualize the current image or image stack under analysis.
    - *Contrast Adjustment*: A pop-up window can enable users to adjust contrast to enhance visualization.
    - *Reset View*: Resets the image zoom to the max outward level.
    - *Package Option*: Provides options for users to select an analysis pipeline, such as *Particle Analysis* or *Chromatic Aberration Calibration* (see **General note 9**).
    - *Start Analysis*: Opens and displays the bounding boxes for detected particles at the selected threshold, offering single or multi-thresholding.
    - *Fit Gaussians*: Performs the 2D/3D Gaussian fitting at the specified threshold and displays the fitted particles with localizations and bounding boxes.
    - *Fitting Options*: Offers a choice between 2D and 3D Gaussian fitting for users. The 2D option is compatible with both 2D and 3D images, providing measurements of detected objects at their optimal focus plane when applied to 3D images.
    - *Match & Align Points*: Performs matching of corresponding fluorescent particles across different channels after their analysis for further examination (see **General note 10**).
    - *Distance Matrices*: Generates distance matrices for every particle set within the same or other channels.
    - *Aberrations*: Facilitates the characterization and generation of polynomial calibration surfaces or affine transformations for calibrating between different channels.
    - *Save Channel Results*: Saves analysis results for the current channel, visualized when switching between channels.
    - *Export Results*: Exports all analysis parameters to an Excel sheet and a folder, including a reference image and aberration calibration.
    **Figure 2.**
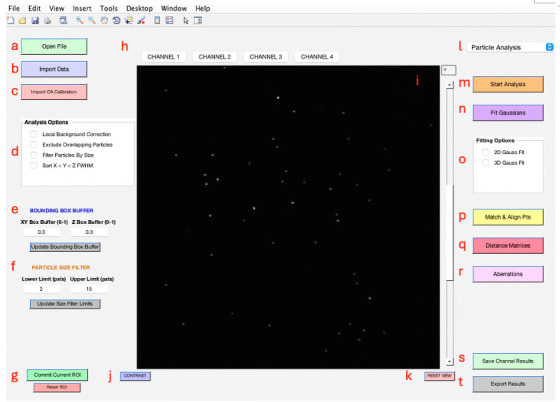
Main interface of 3D-Speckler. This figure displays the primary user interface of the 3D-Speckler software.
  2. Measurements of size below and above PSF, illumination flatness, and chromatic aberration
    - Set the MATLAB working directory to the location that includes the “bfmatlab” folder and the 3D-Speckler script.
    - Use the command “Open3DSpeckler” in the command window to run the 3D-Speckler or click *Run* (see **General note 11**).
    - Select a bead image file in 3D-Speckler. Users can choose image stacks that are either single-wavelength or multi-wavelength (see **General note 12**).
    - For multi-wavelength images, select the appropriate channel wavelengths and adjust the image scales. If users select an image that is single-wavelength, 3D-Speckler will prompt users to decide whether to add more wavelength images (Figure 3) (see **General note 13**).
      **Figure 3.**
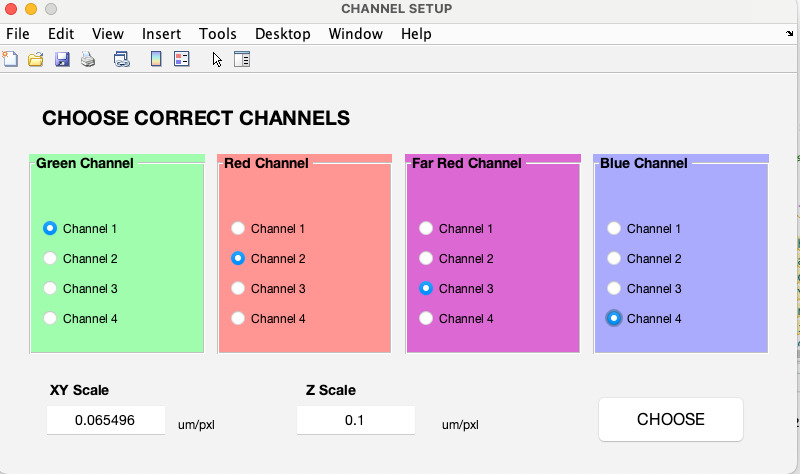
Selecting image wavelengths. Upon selecting an image file, it will be displayed. You will then need to choose the wavelengths and review both lateral and axial scales.
    - The main user interface will launch upon clicking *choose*. After completing this step, the 3D-Speckler base interface will appear (Figure 4), allowing you to select either *Particle Analysis* or *Local Chromatic Aberration Correction* for downstream analysis.
      **Figure 4.**
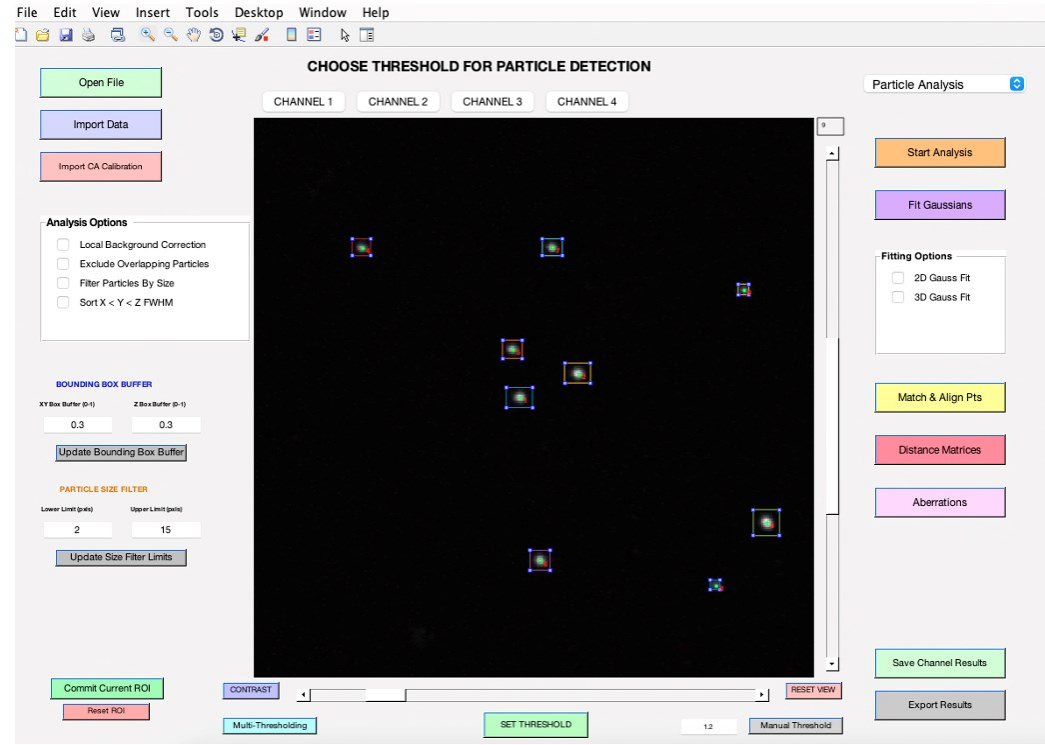
3D-Speckler main interface. After completing wavelength selection, this interface will be displayed. You will need to select either *Particle Analysis* or *Local Chromatic Aberration Correction* for downstream analysis.
    - Choose the *Particle Analysis* pipeline from the top right-hand corner (see **General note 14**).
    - Select your preferred analysis options from the panel on the middle left-hand side (refer to **feature d**). When you choose the *Local Background Correction* option in 3D-Speckler, the software will subtract local background signals from each detected object (see **General note 15).** 3D-Speckler performs local background correction using a user-defined percentage for a larger bounding box (default is 15% larger in all dimensions) with automated BG exception handling. The *Exclude Overlapping Particles* feature ensures that particles with overlapping bounding boxes are removed to avoid measurement inaccuracies. The *Sort by X < Y < Z FWHM* option organizes results in an Excel file based on the full width at half maximum (FWHM) values for the X, Y, and Z axes. Before starting the analysis, confirm the ROI by using the zoom button to select a specific area if you aim to analyze a particular region (refer to **feature g**).
    - To adjust the image contrast, utilize the *Contrast* feature (refer to **feature j**). For Z-position adjustments, slide the bar situated to the right of the image. To choose a specific wavelength, click on the *Channel* feature (refer to **feature h**).
    - Begin by clicking on *Start Analysis* to set the optimal detection threshold (Figure 5). This allows users to view bounding boxes, which can be adjusted according to the thresholding bar or via the bounding box buffer fields (refer to **feature e**). Once the ideal threshold is determined, confirm by clicking *SET THRESHOLD* (see **General note 16**).
      **Figure 5.**
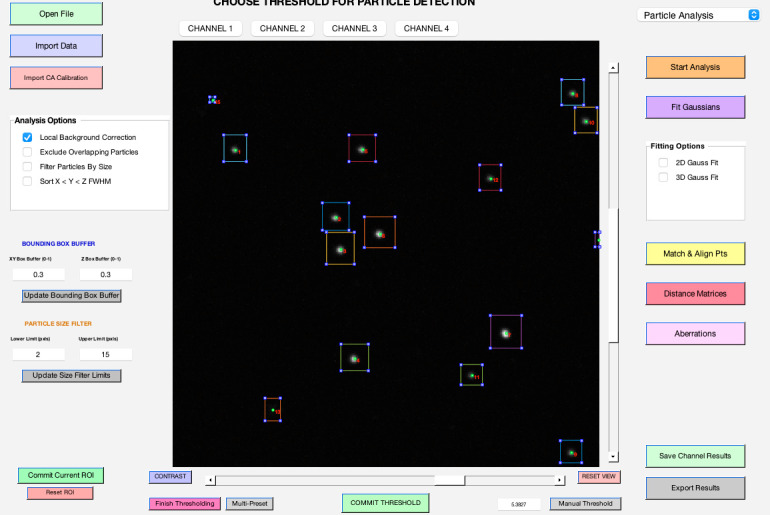
Thresholding bar. It pops up when you click *Start Analysis*. Users can adjust the threshold by sliding the bar to the right or left.
    - Select the fitting options (either *2D Gauss Fit* or *3D Gauss Fit*; refer to **feature o**) and click on *Fit Gaussians* to fit particles.
    - (Optional) If you would like to perform local background correction, you need to select the parameters below (Figure 6) (see **General note 15**).
      **Figure 6.**
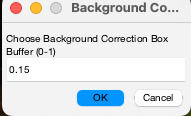
Background correction buffer. This option becomes available after you click on *Fit Gaussians*. If you wish to perform background correction, please select the size of the bounding box for background measurements. Choosing *1* will double the dimensions of the bounding boxes used for quantification (see **General note 15**).
    - Review and manage detected particles: To remove unwanted particles, first click the *Select* button found beneath the *Fit Gaussians* button. Then, within their bounding boxes, double-click on the particles you wish to eliminate (Figure 7). Once you have reviewed the detected objects, click on *Save Channel Results* to save the data for the current channel. Ensure you save your data before moving to the next channel; otherwise, unsaved measurement results will be lost.
      **Figure 7.**
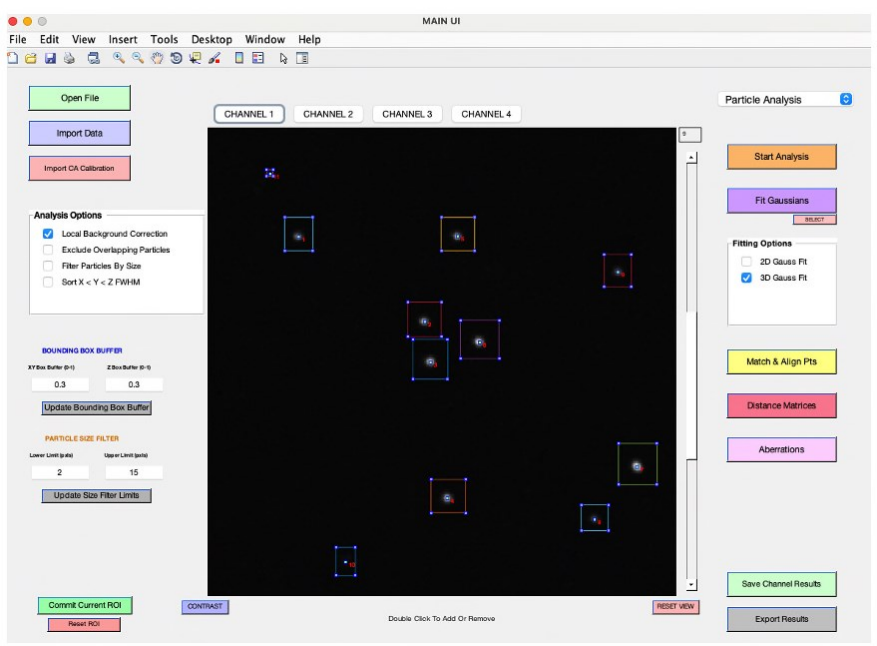
Review fitting results. After fitting, particle locations will be shown in blue, not green. Click the *select* button located under the *Fit Gaussians* button, and then double-click on particles within their bounding box to remove them.
    - Repeat steps B2h–B2l to analyze each channel.
    - After completing the particle analysis for all channels, select *Match & Align Pts* (refer to **feature p**) to match corresponding particles and align all channels (Figure 8). This step is optional for users who only require measurements of size and intensities. The *Match & Align Pts* feature identifies corresponding beads across various wavelengths, enabling the comparison of measurement values from different wavelengths within the same TetraSpeck beads. However, this process will eliminate detected particles that do not match. Utilizing this option is essential for further analysis of chromatic aberrations.
      **Figure 8.**
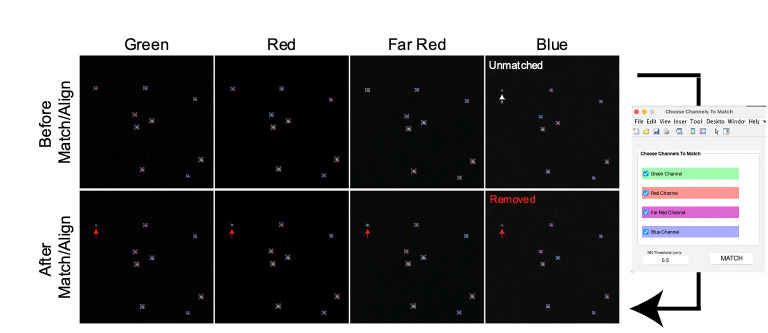
Matching and aligning detected particles across all channels. The *Match & Align Pts* function enables the selection of corresponding particles across different channels, eliminating those that do not match. The white arrow indicates a particle unmatched in the Blue channel, resulting in its removal from quantification in all channels, as shown by the Red arrows.
    - To measure chromatic aberrations, clicking *Aberrations* (refer to **feature r**) will open the wavelength selection tab. Choose the wavelength at which you wish to determine aberrations, and then click *Generate* (see **General note 17**).
    - Select *Export Results* (refer to **feature t**). 3D-Speckler will prompt you to decide if you wish to include *Field Dependence Plots* in your export. If you are interested in assessing the uniformity of illumination, please choose *Yes*. Then, specify the desired location to save the files. Upon doing so, multiple plots will be displayed, followed by a confirmation message from 3D-Speckler indicating “Data Export Completed Successfully.”
  3. Reviewing results
    - 3D-Speckler will create a new folder that includes an Excel file, MATLAB images for each channel, and a subfolder containing field-dependent plots of FWHM and signal intensities. The MATLAB images of beads will feature assigned numbers for each detected bead, corresponding to the numbering in the Excel file.
    - To review and obtain the 2D and 3D size of TetraSpeck beads, open the Excel file (Figure 9). 3D-Speckler offers three distinct methods for measuring FWHM: true (tFWHM), interpolated (iFWHM), and Gaussian FWHM (gFWHM). The differences lie in the methods used to obtain the intensity profile of detected particles for measuring FWHM. Detailed descriptions of these measurements can be found in our original article [18]. Briefly, gFWHM and iFWHM use Gaussian and interpolation fitting methods, respectively, whereas tFWHM does not employ mathematical fitting methods. We recommend using tFWHM for lateral measurements and iFWHM for axial measurements [18]. The average lateral size (tFWHM) can be determined by averaging the values of the blue columns in the Excel file (Column Q and R in an Excel file in 3D measurements). Similarly, the axial size (iFWHM) can be calculated by averaging the values in the black column (Column V in an Excel file in 3D measurements) (see **General note 18**).
      The lateral and axial sizes of beads smaller than the PSF represent the resolution limit of your system (measured PSF in your system). You can compare and evaluate these values against the theoretical resolution limit of your system. The measured size of beads larger than the PSF should be equal to or close to their actual size. If not, it may indicate that the bead size you used is still smaller than the PSF of your system or suggest potential defects in your system.
      **Figure 9.**
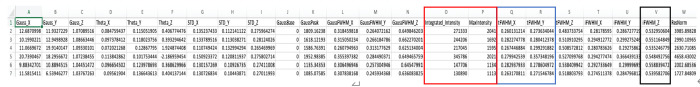
Quantification results. Example results of the green channel. The output is in Excel file format. Abbreviations’ definitions are listed in Table 1.
      **Table 1.** Definition of the abbreviations in Figure 9. Explanation of the abbreviations in the output Excel file.

Abbreviation	Explanation
Gauss_X, _Y, -Z	x, y, and z coordinates of the center position of detected particles.
Theta_X, _Y, _Z	x, y, and z angles based on the optimum center of detected particles, as described in the original 3D-Speckler manuscript.
STD_X, _Y, _Z	Standard of deviation of Gaussian fitting.
GaussBase	The base signal intensity of Gaussian fitting. If users select local background correction option, this value is used for local background correction.
GaussPeak	The peak signal intensity of Gaussian fitting.
tFWHM_X, _Y, _Z	tFWHM values of each detected particle in x, y, and z-axis.
iFWHM_X, _Y, _Z	iFWHM values of each detected particle in x, y, and z-axis.
gFWHM_X, _Y, _Z	gFWHM values of each detected particle in x, y, and z-axis.
ResNorm	Normalized residual value: how well the objects fit the 2D/3D Gaussian model.
    - 3D-Speckler measures integrated and maximum signal intensities for each bead [the red columns (O and P) in Figure 9]. Using this information, 3D-Speckler can generate field dependence plots that illustrate variations in the FWHM measurements and intensity across the FOV of an imaging setup. Users will have the option to export these plots. If chosen, 3D-Speckler will save the plots as .fig files in the *Field Dependence Plots* folder. These plots can then be analyzed to identify areas within the field of view that provide the most accurate measurements (Figure 10).
      **Figure 10.**
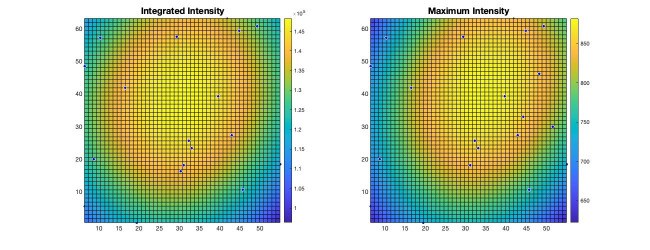
Visualization of field dependency plots. Surface plots of integrated intensity and maximum intensity variations illustrate changes across the imaging field of view (FOV). The light source in fluorescence microscopy is typically brightest at the center of illumination and gradually becomes dimmer toward the edges.
    - When the *Aberrations* case (step B2o) is executed, the Excel file contains chromatic aberration measurements across the selected wavelengths. These results are presented in the worksheet titled “aberration_ wavelength” in the Excel file (Figure 11 and **General note 19**). The file includes aberration data for each axis and calculates the 2D/3D distances for individual beads. To obtain average aberration values and distances, users can average these data points.
      **Figure 11.**
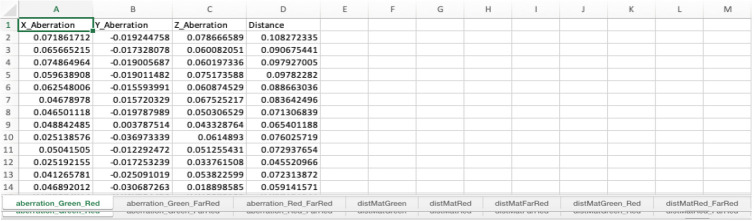
Aberration between different channels. The Excel file contains the worksheets named “aberration_wavelength”. These worksheets include aberration in x, y, and z-axis and 3D distance measurements (µm scale) (see **General note 19-20**).

## Validation of protocol

This protocol or parts of it has been used and validated in the following research article

- Loi, J. et al. (2023). Semi-automated 3D fluorescence speckle analyzer (3D-Speckler) for microscope calibration and nanoscale measurement. J Cell Biol. (Figures 1–8).

## General notes and troubleshooting

**General notes**

1. Any fluorescence bead slide can be utilized; however, we recommend using beads of at least two sizes: one smaller than the PSF (< 200 nm) and one larger than the PSF (e.g., 250 nm, 500 nm, and 1 µm). We observed that the mounted beads on slides tend to shrink over time. Therefore, we advise using fresh beads or those used shortly after purchase. Commercial or homemade bead slides should be stored at 4 °C and protected from light.
2. Fluorescent bead calibration slides can be made by yourself.
3. For users who do not have access to a MATLAB license, we provide a standalone, executable application made through MATLAB compiler. The standalone 3D-Speckler and user manual are available at the following website (Windows: https://drive.google.com/drive/u/3/folders/1iV5AbqgJhIkQV2lAoZyrNN0BRRq5_JrX and Mac: https://drive.google.com/drive/u/3/folders/1wrVNDN-6H0QAMgidj0Ql_XTvt44GGkFR.). Running standalone 3D-Speckler requires installation of the correct version of MATLAB Runtime, thus we provided an installer for the correct version in the same download folder.
4. 3D-Speckler can be installed on both Windows and Mac OS with MATLAB (2019 and above).
5. We recommend obtaining images with high SNR, ideally > 20–50, to calibrate the optimal performance of your system. However, the specific SNR requirements may vary depending on your system. You can test the effects of SNR on your system's performance by preparing bead images with different SNRs, which can be achieved by adjusting the light source power and/or camera exposure time.
6. We recommend imaging with 100 or 200 nm steps.
7. Since calibrating with a single 2D/3D bead image carries risks, we recommend using multiple images taken from several different locations.
8. 3D-Speckler uses the Bio-Formats (OME) software tool, which can open most image file formats [25].
9. *Protein Quantification* is under development and currently unavailable.
10. Unmatched points will be removed from further analysis.
11. Executing 3D-Speckler by clicking *Run* does not require changing the working directory to include the “bfmatlab” folder. However, to use the “Open3DSpeckler” command for operation, it is necessary to change the working directory.
12. 3D-Speckler automatically reads and categorizes images into different channels. If only one single is detected, it allows users to import data for more channels.
13. 3D-Speckler automatically detects the lateral and axial resolution by using the image metadata. If these resolutions are not automatically filled in or the numbers have errors, users must manually input or correct the scaling values.
14. The 3D-Speckler interface automatically updates based on the selected analysis options and workflow. *Particle Analysis* is the default analysis method for analyzing general fluorescent particles.
15. For advanced settings, 3D-Speckler provides a *Local Background Correction* option for signal intensity and Gaussian fitting measurements. Since background signals are not uniform across the FOV, local background correction emerges as one of the most accurate measurement methods [14,15,18]. To utilize this feature, users need to optimize parameters for further analysis. The default is set at 0.15 (which is 15% larger in all dimensions than the bounding box used for measurements in each detected particle to obtain local background signals). For most analyses involving TetraSpeck beads, this default setting proves effective.
16. For an additional optimization step, by clicking on *multi-thresholding*, users can select a threshold to commit an image. 3D-Speckler will then remove those objects, allowing users to set another threshold until all desired objects are accurately detected. Conclude by clicking on *Finish Thresholding*.
17. The *Distance Metrics* option offers 2D or 3D distance measurements between all detected beads, both within a single channel and across different channels [18]. This feature is not necessary for microscope calibration.
18. 3D-Speckler offers three different measurement methods for FWHM. Please see the details in the original article [18].
19. This measurement boasts a precision of less than 5 nm [18].
20. 3D-Speckler provides correction for chromatic aberration and outputs the corrected images, as detailed in the original article [18].
